# QTL mapping of flag leaf-related traits in wheat (*Triticum aestivum* L.)

**DOI:** 10.1007/s00122-017-3040-z

**Published:** 2018-01-23

**Authors:** Kaiye Liu, Hao Xu, Gang Liu, Panfeng Guan, Xueyao Zhou, Huiru Peng, Yingyin Yao, Zhongfu Ni, Qixin Sun, Jinkun Du

**Affiliations:** 1 State Key Laboratory for Agrobiotechnology, China Agricultural University, Beijing, 100193 China; 2 Key Laboratory of Crop Heterosis and Utilization (MOE), China Agricultural University, Beijing, 100193 China; 3 Beijing Key Laboratory of Crop Genetic Improvement, China Agricultural University, Beijing, 100193 China; 4 High School Attached to Captain Normal University, Beijing, 100048 China

## Abstract

**Key message:**

QTL controlling flag leaf length, flag leaf width, flag leaf area and flag leaf angle were mapped in wheat.

**Abstract:**

This study aimed to advance our understanding of the genetic mechanisms underlying morphological traits of the flag leaves of wheat (*Triticum aestivum* L.). A recombinant inbred line (RIL) population derived from ND3331 and the Tibetan semi-wild wheat Zang1817 was used to identify quantitative trait loci (QTLs) controlling flag leaf length (FLL), flag leaf width (FLW), flag leaf area (FLA), and flag leaf angle (FLANG). Using an available simple sequence repeat genetic linkage map, 23 putative QTLs for FLL, FLW, FLA, and FLANG were detected on chromosomes 1B, 2B, 3A, 3D, 4B, 5A, 6B, 7B, and 7D. Individual QTL explained 4.3–68.52% of the phenotypic variance in different environments. Four QTLs for FLL, two for FLW, four for FLA, and five for FLANG were detected in at least two environments. Positive alleles of 17 QTLs for flag leaf-related traits originated from ND3331 and 6 originated from Zang1817. QTLs with pleiotropic effects or multiple linked QTL were also identified on chromosomes 1B, 4B, and 5A; these are potential target regions for fine-mapping and marker-assisted selection in wheat breeding programs.

**Electronic supplementary material:**

The online version of this article (10.1007/s00122-017-3040-z) contains supplementary material, which is available to authorized users.

## Introduction

Flag leaves of wheat (*Triticum aestivum* L.), regarded in crop production as the “functional leaves”, are the main organs for photosynthesis, and contribute 45–58% of photosynthetic performance during the grain-filling stage (Duncan 1971; Khaliq et al. 2008). Morphological traits of the flag leaves are one of the most important determinants of plant architecture and yield potential (Duncan 1971; Guitman et al. 1991; Sharma et al. 2003). For example, Duwayri (1984) found that grain yield and kernel number per plant were reduced when flag leaves were removed.

The size of the flag leaf is estimated by flag leaf length (FLL), width (FLW), and area (FLA), and is positively correlated with the thousand-grain weight (TGW), panicle weight (PW), and other yield-related traits in cereals (Cui et al. 2003; Wang et al. 2010, 2011; Yue et al. 2006). Flag leaf angle (FLANG) determines the amount of incident light that the leaf receives.

Donald (1968) regarded upright leaves as an “ideotype” for wheat. Having vertical leaves improves sunlight capture, thus enhancing photosynthesis and the production of dry matter. For example, plants with erect leaves can produce higher yields compared with plants with lax leaves, given sufficient water (Joshi 1997). Therefore, breeders have used this trait to optimize plant architecture (Angus et al. 1972; Austin et al. 1976; Morinaka et al. 2006).

There have been several efforts to uncover the genetic mechanisms underlying flag leaf morphology in wheat. The early studies showed that flag leaf size and angle are complex quantitative traits that are controlled by many genes or quantitative trait loci (QTLs), and are significantly influenced by the environment (Simon 1999). Putative QTLs with flexible expression in various genetic populations and environments have been detected on almost all 21 wheat chromosomes. For example, in durum wheat, FLANG, FLL, and FLW were mapped to chromosomes 2A, 3B, 5B, 7A, and 2B (Isidro et al. 2012). A major QTL for FLW was fine-mapped into an interval of 0.2 cM, between markers *Xwmc492*–*Xwmc752*, on chromosome 5A (Xue et al. 2013). Using a recombinant inbred line (RIL) population with an integrated high-density simple sequence repeat (SSR) and single-nucleotide polymorphism (SNP) genetic linkage map, 17 QTLs for FLANG, 11 for FLW, 7 for FLL, 13 for FLL:FLW ratio (FLR), and 13 for FLA were identified (Wu et al. 2015). Thirty-eight QTLs for FLW, FLL, and FLA were identified in eight environments, among them two stable QTLs for FLL on chromosomes 4B and 6B, and one for FLA on chromosome 5B (Fan et al. 2015). Twenty stable QTLs for flag leaf morphology are potentially useful for genetic improvement of drought tolerance in wheat through QTL pyramiding (Yang et al. 2016).

This study aimed to (1) identify QTL regions linked to flag leaf morphology such as FLL, FLW, FLA, and FLANG; and (2) understand their effect on grain yield-related traits using an RIL population developed from a cross between a locally adapted and semi-wild wheat parent lines. The results of this study provide a better understanding of the genetic mechanisms controlling flag leaf-related traits, and might be helpful for the genetic improvement of wheat plant architecture and yield potential.

## Materials and methods

### Plant materials and field trials

The mapping population, which consisted 213 RILs, was derived from a cross between the locally adapted line ‘ND3331’ and a Tibetan semi-wild wheat ‘Zang1817’. After the initial cross, the lines were advanced until the F_9_ generation using single seed descent (Liu et al. 2014). Compared to ND3331, the Zang1817 had smaller and more erect flag leaves, poorer yield traits and stronger biotic and abiotic stress resistance/tolerance. The RILs together with the parent lines were planted in Beijing (39°57ʹN, 116°17ʹE) in 2015 and 2016 (2015-BJ and 2016-BJ), and Shijiazhuang (38°03ʹN, 114°26ʹE), Hebei province (2016-HB), Taiyuan (36°16ʹN, 108°04ʹE), Shanxi province (2016-SX), and Xinxiang (35°18ʹN, 113°55ʹE), Henan province (2016-HN) in 2016. In each environment, seeds were planted in three completely randomized blocks, each block with 200 cm-long rows spaced 30 cm apart with a sowing rate of 30 seeds per row. All field trials were well watered and managed in accordance with standard local practices.

### Testing flag-related traits and statistical analysis

At flowering stage, main tillers of ten representative plants from each RIL were used for flag leaf phenotypic evaluation. FLL was measured as the distance from the base to the tip of the leaf; FLW as the width of the widest section of the leaf; and FLANG as the angle between the stem immediately below the spike and the flag leaf midrib, with more upright leaves having a smaller leaf angle. FLA, a derived trait, was defined as FLL × FLW × 0.75 (Edae and Byrne 2013; Yang et al. 2016). The phenotype data of six yield-related traits including plant height (PH); spike length (SL); spike number per plant (SN); kernel number per spike (KN); kernel weight per spike (KW); thousand-grain weight (TGW) from 2008 to 2010 in Beijing and from 2008 to 2009 in Shanxi were obtained from Liu et al. (2014).

For each RIL individual, standard deviation for each phenotypic value among main tillers of ten representative plants was calculated. For individuals with a large standard deviation; the maximum and minimum values were eliminated and the remainders were used to calculate mean. The skewness and kurtosis were calculated using “SKEW” and “KURT” functions in Microsoft Office Excel. The two-tailed Student’s *t* test was used for detecting differences in parental phenotypes. Broad-sense heritability was calculated using the PROC GLM procedure in SAS (SAS Institute, Cary, NC, USA), based on the following formula:

$$ H^{2} = V_{\text{G}} /(V_{\text{G}} + V_{\text{E}} ) \times 100\% , $$

where *V*_G_ is genetic variance and *V*_E_ is environmental variance.

Correlations analysis was between pairs of flag leaf-related traits and yield-related traits in the RIL population were performed by Pearson correlation in the SAS software. Adjusted mean values (best linear unbiased predictions, BLUP) (Supplement Table 1) across evaluated environments were calculated using the PRO MIXED procedure in SAS and used for correlations analysis. The BLUP value for flag leaf-related traits was calculated as follows: *y*_*ij*_ = *u* + *E*_*i*_ + *G*_*j*_ + *ε*_*ij*_, where *y*_*ij*_ is the phenotypic value of individual *j* in the environment *i*, *u* is the grand mean for all environments, *E*_*i*_ is the effect of different environments, *G*_*j*_ is the genetic effect, and *ε*_*ij*_ is the random effect. The grand mean was fitted as a fixed effect, and genotype and environment were considered as random effects (Wang et al. 2015).

### QTL analysis

A previously published whole-genome genetic linkage map was used, which contains 335 polymorphic markers and spans 2994.5 cM, with an average spacing of 9.4 cM (Liu et al. 2014). Map distances were converted from recombination frequencies using the Kosambi mapping function (1944), and JoinMap4.0 software was used to create the genetic linkage map (Van Ooijen 2006). The Map Draw (V2.1) software was used to draw the map (Liu and Meng 2003). Averaged trait values for plants grown in each environment were used for QTL analysis, and BLUP across five environments were used for combined analysis. Windows QTL Cartographer (v2.5) software was used for composite interval mapping (CIM) to identify and analyze QTL (Wang et al. 2007). Using this method, limit-of-detection (LOD) scores were calculated with 1000 permutations at *P* ≤ 0.05, and LOD ≥ 2.5 indicative of a QTL. *R*^2^ was estimated as the percentage of variance explained by each locus in proportion to the total phenotypic variance.

For conditional QTL analysis, the conditional phenotypic values (T1|T2) (Supplemental Table 2), which means value of Trait1 conditional on Trait2, were calculated using QGAStation2_0.exe software (Chen et al. 2012). Windows QTL Cartographer (v2.5) software was used to identify the conditional QTL with conditional phenotypic values.

## Results

### Phenotypic variation of flag leaf-related traits

**Fig. 1:**
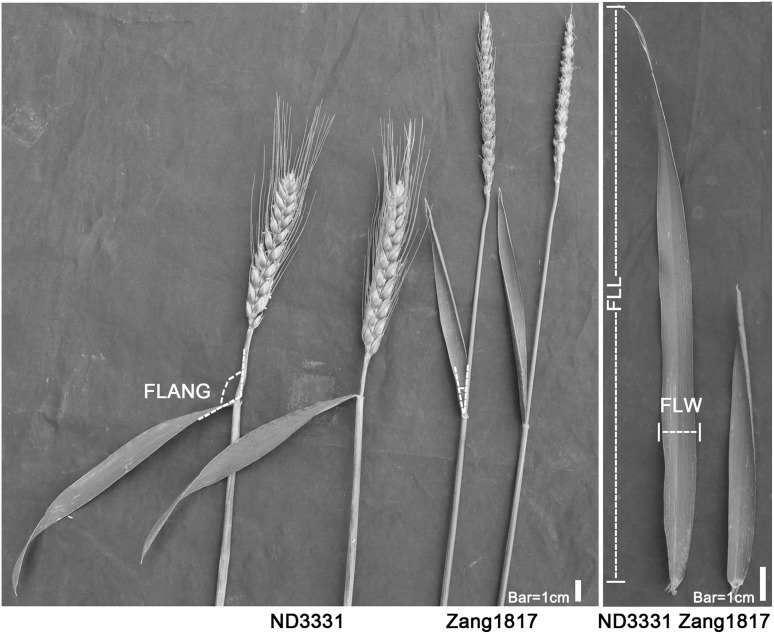
Morphology of the flag leaves of ND3331 and Zang1817. Flag leaves of ND3331 and Zang1817 at flowering stage from the Beijing 2015 trail. Flag leaf length (FLL) is the distance from the base to the tip of the flag leaf. Flag leaf width (FLW) is the width of the widest section of the flag leaf. Flag leaf angle (FLANG) is the angle between the stem immediately under the spike and the flag leaf midrib. Bar 1 cm

**Fig. 2:**
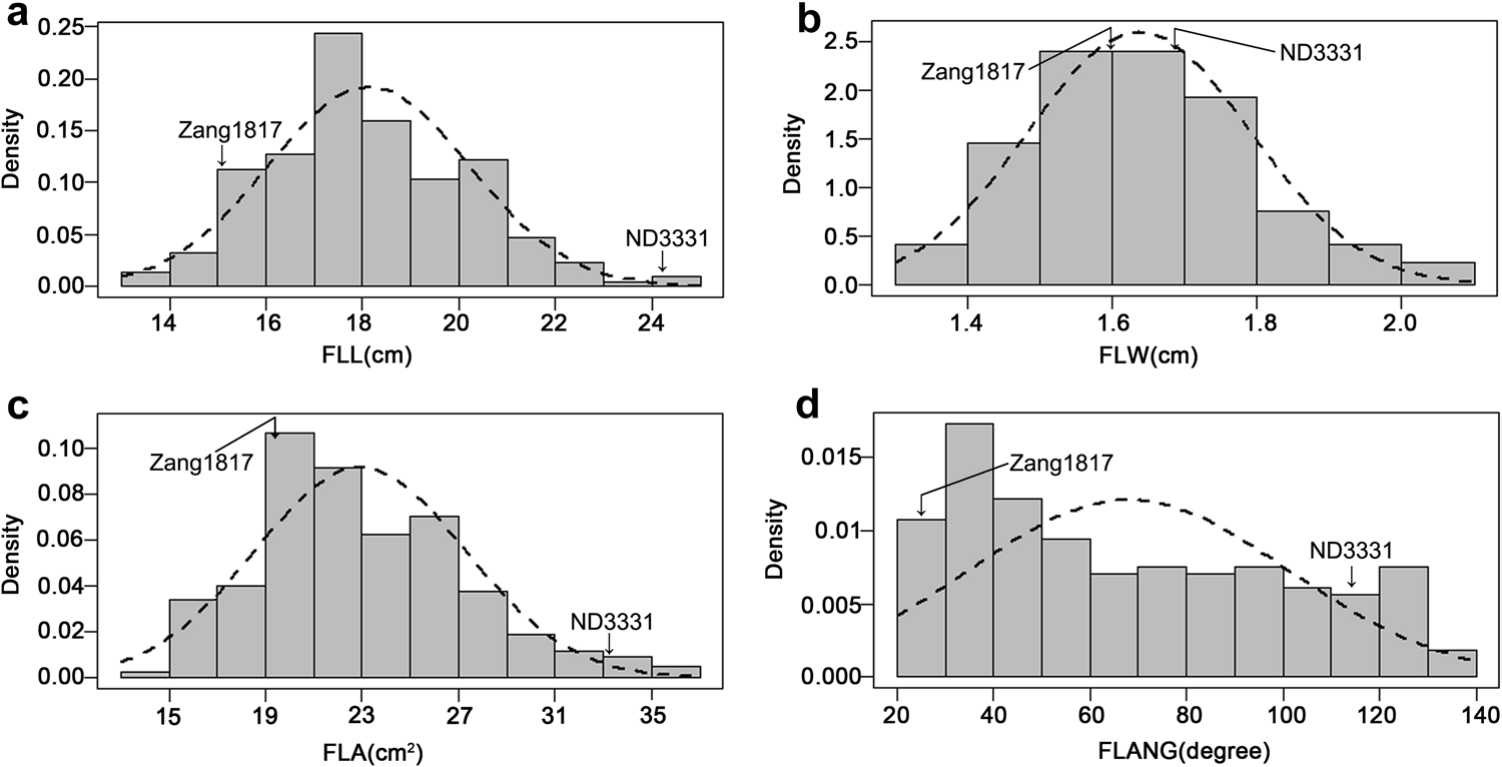
Histograms of flag leaf-related traits in the ND3331 and Zang1817 recombinant inbred population. **a** Flag leaf length (FLL); **b** flag leaf width (FLW); **c** flag leaf area (FLA); **d** flag leaf angle (FLANG)

Table 1 summarizes the phenotypic means for flag leaf-related traits, including FLL, FLW, FLA, and FLANG, from the parent and RIL populations, and basic statistics from plants grown in five environments. The two parent lines, ND3331 and Zang1817, were significantly different in terms of all investigated traits (except FLW). In ND3331, phenotypic means for FLL, FLW, FLA, and FLANG were higher than those of Zang1817 in all environments (Table 1, Fig. 1). In the RIL population, bidirectional transgressive segregation was observed for all four traits, and the frequency distribution of FLL, FLW, and FLA showed continuous variation. The skewness and kurtosis for FLL, FLW (except 2015-BJ), and FLA (except 2015-BJ and 2016-BJ) indicated that a normal distribution of these traits. However, variation in FLANG did not show a normal distribution in four environments (Fig. 2). The broad-sense heritability of FLL, FLW, FLA, and FLANG were 83, 88, 79, and 82%, respectively (Table 1).

**Table 1:** Phenotypic performance and distribution of flag leaf-related traits in parent and RIL populations

Trait	Environment	ND3331	Zang1817	RIL				
		Mean	Mean	Mean ± SD	Range	Skewness	Kurtosis	*H*^2^ (%)
FLL (cm)	2015-BJ	20.68	11.80**	14.9 ± 3.21	7.97–25.9	0.76	0.50	83
	2016-BJ	23.35	14.90**	17.4 ± 3.06	11.63–27.87	0.83	0.62	
	2016-SX	25.33	14.96**	18.38 ± 2.81	10.62–26.73	− 0.05	0.28	
	2016-HB	32.40	19.25**	22.46 ± 3.37	14–31.27	0.13	− 0.23	
	2016-HN	24.42	15.48**	17.37 ± 3.76	10.33–29.7	0.74	0.40	
FLW (cm)	2015-BJ	1.48	1.40*	1.44 ± 0.2	0.98–2.33	0.85	2.27	88
	2016-BJ	1.73	1.71	1.67 ± 0.17	1.25–2.23	0.36	− 0.13	
	2016-SX	1.67	1.64	1.65 ± 0.2	1.03–2.22	− 0.09	0.20	
	2016-HB	1.80	1.70*	1.81 ± 0.2	1.37–2.52	0.38	0.22	
	2016-HN	1.62	1.57	1.6 ± 0.18	1.27–2.15	0.57	0.14	
FLA (cm^2^)	2015-BJ	24.18	13.14**	16.39 ± 5.44	7.07–43.78	1.49	3.71	79
	2016-BJ	31.91	20.13**	22.05 ± 5.54	12.07–43.61	1.02	1.04	
	2016-SX	33.44	19.41**	22.91 ± 5.05	9.8–39.1	0.05	0.20	
	2016-HB	46.07	25.85**	30.71 ± 6.84	14.88–53.67	0.53	0.70	
	2016-HN	30.48	19.57**	21.18 ± 6.44	10.51–45.55	0.94	0.91	
FLANG (degree)	2015-BJ	126.83	19.23**	64.74 ± 46.38	13.73–155.73	0.77	− 1.10	82
	2016-BJ	118.76	19.21**	75.96 ± 44.91	13.47–147.95	0.08	− 1.70	
	2016-SX	95.06	17.43**	54.12 ± 39.62	13.94–146.61	0.98	− 0.64	
	2016-HB	129.89	30.99**	69.89 ± 48.42	14.64–156.04	0.43	− 1.64	
	2016-HN	110.89	42.63**	73.14 ± 44.93	13.87–145.36	0.25	− 1.67	

All the data is the average of the phenotypes in each environment

*RIL* recombinant inbred line, *FLL* flag leaf length, *FLW* flag leaf width, *FLA* flag leaf area, *FLANG* flag leaf angle, *BJ* Beijing, *SX* Shanxi, *HB* Hebei, *HN* Henan

*Significance level at *P* = 0.05; **significance level at *P* = 0.01

### Correlation analysis for flag leaf-related traits and yield-related traits

The correlation coefficients between the four flag leaf-related traits and yield-related traits were estimated and listed in Table 2. FLL strongly positively correlated with FLW, FLA, and FLANG (Table 2). FLW significantly and positively correlated with FLA (Table 2). FLA strongly positively correlated with FLANG (Table 2). We analyzed the relationship between flag leaf-related traits and yield-related traits including plant height (PH), spike length (SL), spike number per plant (SN), kernel number per spike (KN), kernel weight per spike (KW), and thousand grains weight (TGW). We found that FLL, FLW and FLA were strongly positively correlated with SL, KN, and KW, and negatively correlated with SN. FLANG was significantly and positively correlated with KW and TGW, while negatively correlated with SN (Table 2).

**Table 2:** Coefficients of pairwise Pearson correlations between flag leaf-related traits and yield-related traits in the RILs population

Traits	FLL	FLW	FLA	FLANG	PH	SL	SN	KN	KW	TGW
FLL	1									
FLW	0.50**	1								
FLA	0.90**	0.82**	1							
FLANG	0.33**	0.08	0.25**	1						
PH	0.01	− 0.16*	− 0.07	− 0.04	1					
SL	0.17*	0.20**	0.21**	0.06	0.21**	1				
SN	− 0.27**	− 0.50**	− 0.44**	− 0.15*	0.18**	− 0.11	1			
KN	0.53**	0.55**	0.61**	0.13	− 0.04	0.31**	− 0.41**	1		
KW	0.36**	0.34**	0.40**	0.29**	0.14*	0.12	− 0.50**	0.50**	1	
TGW	− 0.04	− 0.05	− 0.05	0.25**	0.17*	− 0.11	− 0.26**	− 0.29**	0.61**	1

*FLL* flag leaf length, *FLW* flag leaf width, *FLA* flag leaf area, *FLANG* flag leaf angle, *PH* plant height, *SL* spike length, *SN* spike number per plant, *KN* kernel number per spike, *KW*, kernel weight per spike, *TGW* thousand grains weight

*Significance level at *P* < 0.05; **significance level at *P* < 0.01

### QTL analysis of flag leaf-related traits

**Fig. 3:**
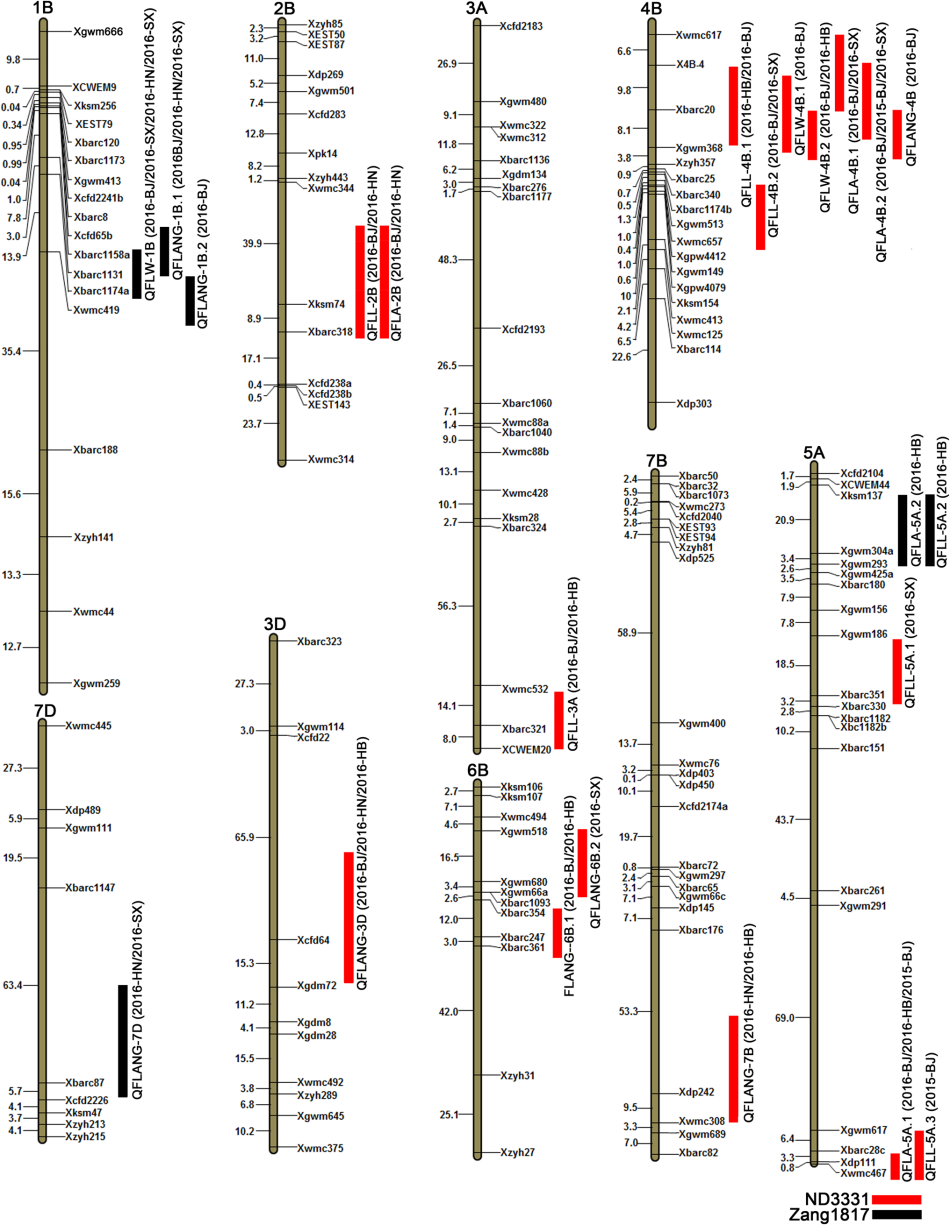
Distribution of quantitative trait loci (QTLs) identified in five environments. *FLL* flag leaf length, *FLW* flag leaf width, *FLA* flag leaf area, *FLANG* flag leaf angle. Map distances (cM) are indicated on the leaf of each chromosome, and marker names are on the right. Limit-of-detection (LOD) peak of each QTL is indicated by a column; red columns show the QTL was contributed by an ND3331 allele, black columns show it was contributed by a Zang1817 allele. Genetic linkage maps were constructed using the software JoinMap 4.0 and MAP Draw V2.1

Twenty-three QTLs for flag leaf-related traits were detected on chromosomes 1B, 2B, 3A, 3D, 4B, 5A, 6B, 7B, and 7D in the five environments. Fifteen QTLs were identified in more than one environment, these QTLs were located on 13 non-overlapping chromosome regions, and eight QTLs were detected just in one environment (Table 3, Fig. 3). These QTLs contributed between 4.3 and 68.52% of the phenotypic variance in different environments (Table 3). ND3331 alleles contributed 17 of the 23 QTLs, and 6 were contributed by Zang1817 alleles (Table 3, Fig. 3).

**Table 3:** QTLs for flag-related traits detected in all environments in the ND3331 and Zang1817 RIL population

Trait	QTL	Environment	Position	Left marker	Right marker	LOD	Add	*R*^2^ (%)
FLL	*QFLL*-*2B*	2016-BJ	52.60	*Xbarc318*	*Xwmc344*	4.31	0.96	9.63
		2016-HN	50.60	*Xbarc318*	*Xwmc344*	5.40	1.31	12.11
	*QFLL*-*3A*	2016-BJ	14.00	*Xcwem20*	*Xwmc532*	2.88	0.74	5.73
		2016-HB	16.00	*Xcwem20*	*Xwmc532*	2.72	0.86	6.38
	*QFLL*-*4B.1*	2016-HB	23.40	*X4B*-*4*	*Xgwm368*	3.35	1.35	8.38
		2016-BJ	25.50	*Xbarc20*	*Xzyh357*	8.66	2.52	9.49
	*QFLL*-*4B.2*	2016-BJ	31.00	*Xbarc340*	*Xgwm513*	2.93	2.07	5.87
		2016-SX	33.30	*Xgwm513*	*Xgpw4412*	3.72	0.90	7.73
	*QFLL*-*5A.1*	2016-SX	143.80	*Xbarc330*	*Xgwm186*	2.65	0.61	4.62
	*QFLL*-*5A.2*	2016-HB	189.50	*Xgwm293*	*Xksm137*	3.54	− 0.90	7.04
	*QFLL*-*5A.3*	2015-BJ	0.80	*Xwmc467*	*Xbarc28c*	3.52	0.83	6.47
FLW	*QFLW*-*1B*	2016-BJ	93.90	*Xbarc1174a*	*Xbarc1158a*	3.72	− 0.04	6.03
		2016-SX	93.90	*Xbarc1174a*	*Xbarc1158a*	3.28	− 0.05	5.90
		2016-HB	93.90	*Xbarc1174a*	*Xbarc1158a*	3.42	− 0.05	6.07
		2016-HN	93.90	*Xbarc1174a*	*Xbarc1158a*	3.42	− 0.05	6.03
	*QFLW*-*4B.1*	2016-BJ	20.40	*X4B*-*4*	*Xgwm368*	5.28	0.07	14.7
	*QFLW*-*4B.2*	2016-BJ	26.50	*Xbarc20*	*Xzyh357*	5.23	0.07	12.32
		2016-HB	26.50	*Xbarc20*	*Xzyh357*	3.45	0.06	8.72
FLA	*QFLA*-*2B*	2016-BJ	50.60	*Xbarc318*	*Xwmc344*	2.79	1.29	5.36
		2016-HN	50.60	*Xbarc318*	*Xwmc344*	3.69	1.85	7.92
	*QFLA*-*4B.1*	2016-BJ	7.00	*Xwmc617*	*Xbarc20*	9.38	2.53	19.27
		2016-SX	8.00	*Xwmc617*	*Xbarc20*	3.13	1.37	6.84
	*QFLA*-*4B.2*	2016-BJ	13.30	*X4B*-*4*	*Xgwm368*	9.79	2.34	16.46
		2016-SX	14.4	*X4B*-*4*	*Xgwm368*	4.43	1.85	7.81
		2015-BJ	18.3	*X4B*-*4*	*Xgwm368*	5.70	1.95	11.78
	*QFLA*-*5A.1*	2016-BJ	0.00	*Xwmc467*	*Xdp111*	2.87	1.17	4.31
		2016-HB	0.00	*Xwmc467*	*Xdp111*	2.68	1.48	4.51
		2015-BJ	0.80	*Xwmc467*	*Xbarc28c*	3.02	1.29	5.59
	*QFLA*-*5A.2*	2016-HB	191.50	*Xgwm293*	*Xksm137*	3.24	− 1.80	6.76
FLANG	*QFLANG*-*1B.1*	2016-BJ	101.70	*Xbarc1131*	*Xcfd65b*	5.76	− 14.79	9.72
		2016-HN	101.70	*Xbarc1131*	*Xcfd65b*	3.21	− 11.38	5.70
		2016-HB	101.70	*Xbarc1131*	*Xcfd65b*	3.48	− 12.15	5.62
		2016-SX	101.70	*Xbarc1131*	*Xcfd65b*	5.80	− 17.26	9.84
	*QFLANG*-*1B.2*	2016-BJ	90.90	*Xwmc419*	*Xbarc1131*	3.48	− 11.75	6.05
	*QFLANG*-*3D*	2016-BJ	98.80	*Xgdm72*	*Xcfd22*	25.47	40.64	62.56
		2016-HN	96.80	*Xgdm72*	*Xcfd22*	34.29	41.00	68.49
		2016-HB	100.80	*Xgdm72*	*Xcfd22*	48.64	47.27	65.52
	*QFLANG*-*4B*	2016-BJ	26.50	*Xbarc20*	*Xzyh357*	2.66	11.60	6.34
	*QFLANG*-*6B.1*	2016-BJ	80.10	*Xbarc361*	*Xbarc354*	4.50	13.44	8.80
		2016-HB	80.10	*Xbarc361*	*Xbarc354*	5.93	16.59	11.51
	*QFLANG*-*6B.2*	2016-SX	100.20	*Xgwm66a*	*Xgwm518*	4.95	13.55	11.43
	*QFLANG*-*7B*	2016-HN	47.70	*Xwmc308*	*Xbarc176*	33.97	40.83	68.52
		2016-HB	45.70	*Xwmc308*	*Xbarc176*	50.21	47.26	65.52
	*QFLANG*-*7D*	2016-HN	51.60	*Xcfd2226*	*Xbarc1147*	31.84	− 40.67	68.34
		2016-SX	49.60	*Xcfd2226*	*Xbarc1147*	22.11	− 37.97	61.38

*LOD* maximum-likelihood LOD score for the QTLs, *Add* ± additive effect. Positive value indicates a positive effect of ND3331, whereas negative value indicates a positive effect of Zang1817, *R*^*2*^*(%)* phenotypic variation explained by the QTL, *FLL* flag leaf length, *FLW* flag leaf width, *FLA* flag leaf area, *FLANG* flag leaf angle, *Position* the distance of the peak LOD value from the left marker

Seven QTLs for FLL were detected; four of these, located on chromosomes 2B, 3A, and 4B, were stable QTL detected in two environments (Table 3). QTLs on chromosomes 2B and 3A were mapped to the intervals *Xbarc318*–*Xwmc344* and *Xcwem20*–*Xwmc532*, respectively. Two adjacent QTLs (*QFLL*-*4B.1* and *QFLL*-*4B.2*) had peak positions at 23.4 and 33.3 cM, respectively, on chromosome 4B. Three QTLs on chromosome 5A were detected only in one environment. These seven QTLs explained 5.87–12.11% of the phenotypic variance (Table 3). Additive effects values indicated that six of the FLL-increasing QTLs were contributed by ND3331 alleles, and one (*QFLL*-*5A.2*) was contributed by Zang1817 alleles (Table 3).

For FLW, three QTLs were mapped to chromosomes 1B and 4B, with individual QTL contributing 6.06–14.7% of the phenotypic variance (Table 3, Fig. 3). In four environments, the QTL *QFLW*-*1B* was detected in the interval *Xbarc1174a*–*Xbarc1158a.* Two nearby QTL, *QFLW*-*4B.1* and *QFLW*-*4B.2*, were detected on chromosome 4B in the similar location interval as QTL for FLL. Alleles from ND3331 contributed to increasing FLW at the *QFLW*-*4B.1* and *QFLW*-*4B.2* loci, and alleles from Zang1817 contributed to increasing FLW at the *QFLW*-*1B* locus (Table 3, Fig. 3).

For FLA, five QTLs were identified on chromosomes 2B, 4B, and 5A; four of which were detected at least two environments. These QTLs explained 4.31 to 19.27% of the phenotypic variation in different environments (Table 3, Fig. 3). Two QTLs were detected on chromosome 4B; of these, *QFLA*-*4B.2* mapped to the *X4B*-*4*–*Xgwm368* interval in three environments. Two QTLs were found on chromosome 5A, of which *QFLA*-*5A.1* was delimited to the markers *Xwmc467* and *Xbarc28c* in three environments. Additive effects implied that all loci contributing to increase FLA came from ND3331 alleles.

For FLANG, eight QTLs were found on chromosomes 1B, 3B, 4D, 6B, 7B, and 7D, with the ratio of phenotypic variation contribution ranging from 5.62 to 68.52% (Table 3, Fig. 3). Five QTLs (*QFLANG*-*1B.1*, *QFLANG*-*3D*, *QFLANG*-*6B.1*, *QFLANG*-*7B*, and *QFLANG*-*7D*) were detected in at least two environments. Three major QTLs mapped to the intervals *Xgdm72*–*Xcfd22*, *Xwmc308*–*Xbarc176*, and *Xcfd2226*–*Xbarc1147* on chromosomes 3D, 7B, and 7D, respectively, with each locus explaining more than 60% of the phenotypic variance. Five FLANG-increasing QTLs including *QFLANG*-*3D*, *QFLANG*-*4B*, *QFLANG*-*6B.1*, *QFLANG*-*6B.2*, and *QFLANG*-*7B* came from ND3331, while *QFLANG*-*1B.1*, *QFLANG*-*1B.2,* and *QFLANG*-*7D* were contributed by Zang1817 (Table 3, Fig. 3).

We found several co-localized regions for flag leaf traits. The QTLs detected for FLL (*QFLL*-*4B.1* and *QFLL*-*4B.2*), FLW (*QFLW*-*4B.1* and *QFLW*-*4B.2*), FLA (*QFLA*-*4B.1* and *QFLA*-*4B.2*), and FLANG (*QFLANG*-*4B*) were co-localized on chromosome 4B. The QTLs for FLL (*QFLL*-*2B*, *QFLL*-*5A.2*, and *QFLL*-*5A.3*) co-localized with FLA (*QFLA*-*2B*, *QFA*-*5A.1,* and *QFLA*-*5A.2*) were also detected on chromosome 2B and 5A. Similarly, QTLs for FLANG (*QFLANG*-*1B.1* and *QFLANG*-*1B.2*) were detected with QTL for FLW (*QFLL*-*1B*) on chromosome 1B.

### Conditional QTL analysis among flag leaf-related traits and yield-related traits

**Fig. 4:**
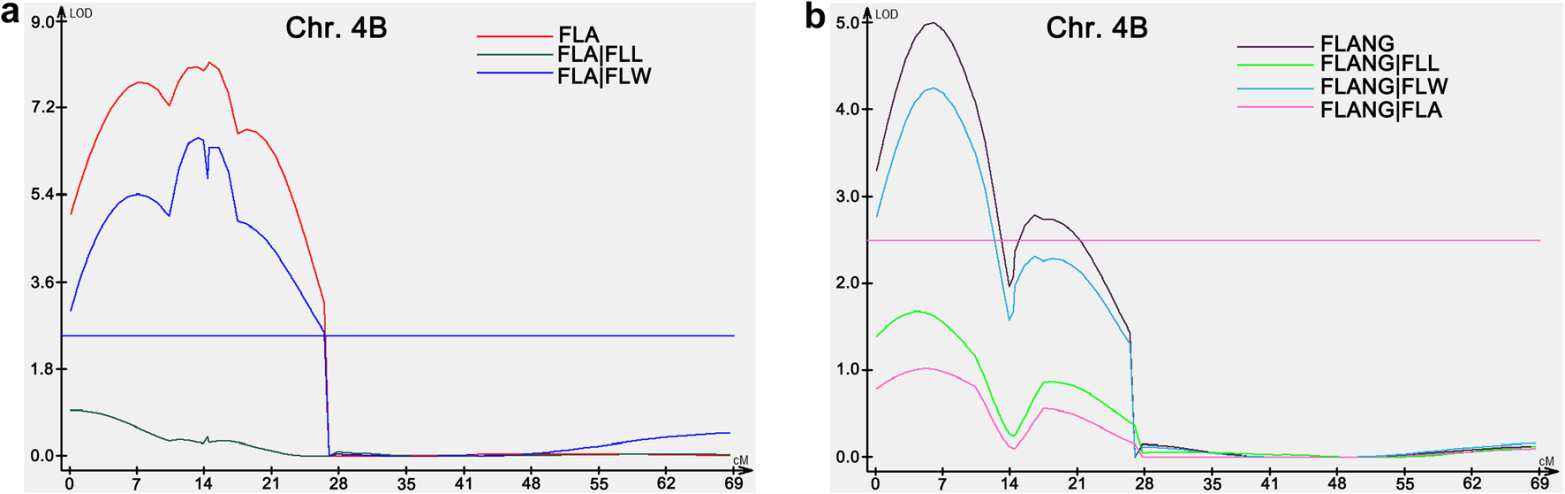
Conditional QTL analysis among flag leaf-related traits. **a** FLA trait conditional on FLL and FLW traits. **b** FLNAG trait conditional on FLL, FLW, and FLA traits

To determine the genetic association between flag leaf-related yield-related traits, conditional QTL analysis was performed. For flag leaf-related traits, when FLA was conditional on FLL, LOD score of *QFLA*-*4B.1* and *QFLA*-*4B.2* was significantly reduced. When FLANG were conditional on FLA and FLL, LOD score of *QFLANG*-*4B.1* locus significantly decreased (Fig. 4). These results indicated that FLL is primarily responsible for FLA and FLANG, and FLA is primarily responsible for FLANG on chromosome 4B locus.

**Fig. 5:**
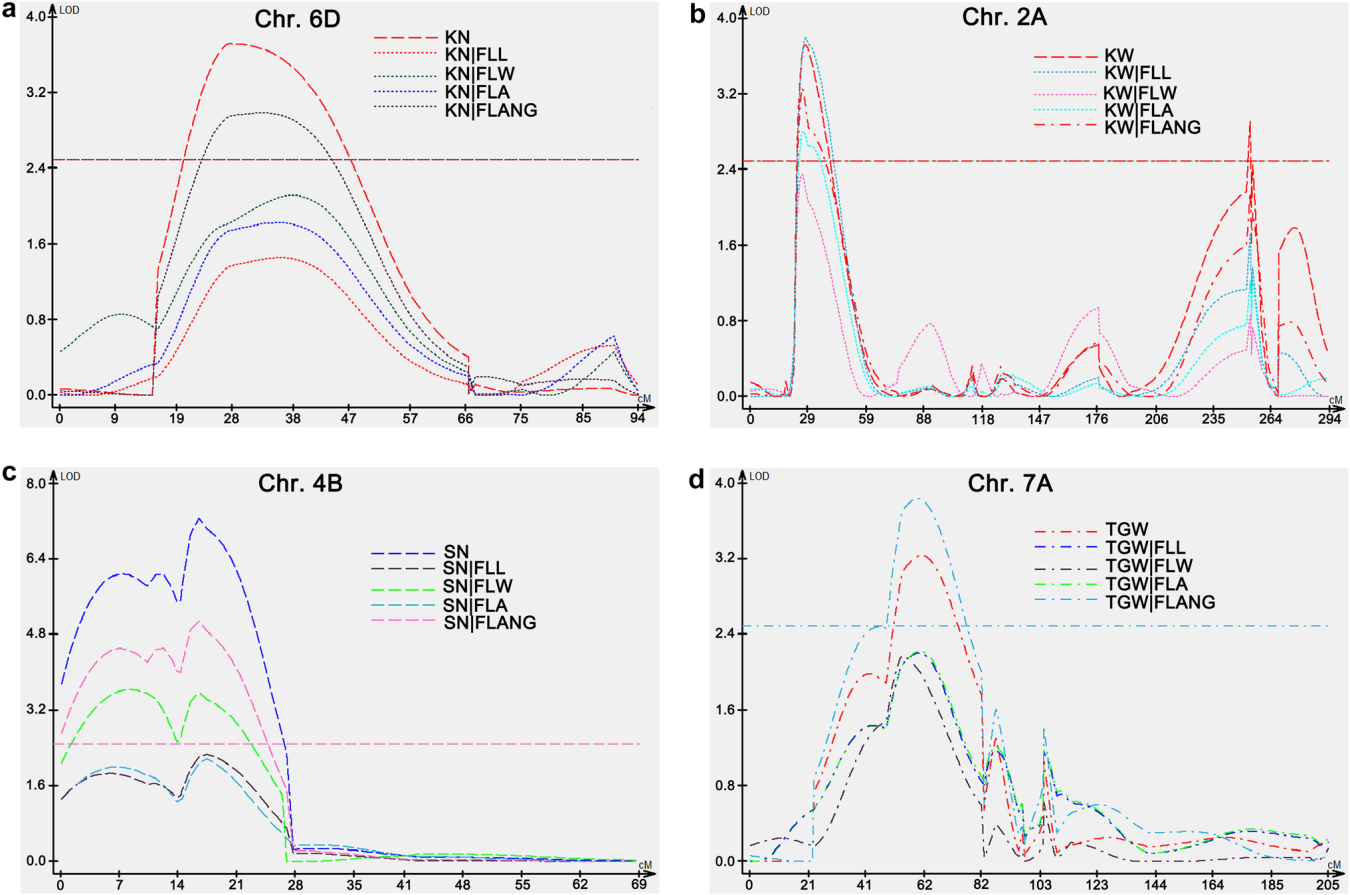
Conditional QTL analysis between flag leaf-related traits and yield-related traits. T1|T2 means Trait1 conditional on Trait2. **a** Kernel number per plant (KN) conditional on FLL, FLW, FLA, and FLANG traits. **b** Kernel weight per plant (KW)conditional on FLL, FLW, FLA, and FLANG traits. **c** Spike number per plant (SN) conditional on FLL, FLW, FLA, and FLANG traits. **d** Thousand grain weight (TGW) conditional on FLL, FLW, FLA, and FLANG traits

When yield-related traits were conditioned on flag leaf-related traits, we found that LOD scores of QTL for KN on 6B and QTL for SN on 4B were significantly reduced when they were conditional on all of flag leaf-related traits. For the QTL for KW on chromosome 2A, the LOD scores were reduced when it was conditional on FLW, FLA, and FLANG (Fig. 5). For the QTL for TGW on 7A, the LOD scores were significantly reduced when it is conditional on FLL, FLW, and FLA, while significantly increased when it is conditional on FLANG (Fig. 5). The results indicated that flag leaf-related traits were responsible for yield-related traits at these loci.

## Discussion

Flag leaf plays important roles in wheat growth and development, and the improvement of flag leaf posture and size has been an important objective in wheat breeding programs. In this study, we analyzed the relationship between flag leaf-related traits and yield-related traits by Person correlation. We found that FLL, FLW, and FLA were strongly positively correlated with spike length, kernel number, and weight per spike, and negatively correlated with spike numbers per plant (Table 2). These results indicated that larger flag leaves contribute to the yield-related traits putatively through providing more photosynthetic products for the spikes and kernels. We also found that FLANG was significantly and positively correlated with KW and TGW, while negatively correlated with SN, which looked inconsistent with the ideal plant architecture with erect flag leaf. We speculated that in ND3331 × Zang1817 population, the significant positive correlation between FLANG and yield-related trait might be resulted from the FLA. One possibility is that FLANG is positively correlated with FLA, indicating the flag leaf angle tended to larger when the flag leaf became bigger which the weight of flag leaf was increased. The second is that according to the conditional QTL analysis (Fig. 5), we found that the FLA has more significant effect responsible for yield-related traits (including KN, KW, SN, and TGW) than FLANG.

The narrow genetic basis of commonly available parental materials remains problematic for wheat breeding programs because of the founder effect. Exploitation of favorable alleles from wild relatives can provide new genetic diversity for wheat breeding, since wild relatives are thought to contain wider genetic diversity than domesticated wheat (Ladizinsky 1985). For example, wild relatives of wheat have been extensively employed in wheat breeding as a source of disease resistance (Sheng et al. 2012; Xie et al. 2011). Tibetan semi-wild wheat is a primitive hexaploid wheat, and is a close relative of wild progenitors of common wheat. In our study, we showed that the Tibetan semi-wild wheat Zang1817 had smaller and more erect flag leaves in all tested environments, which is the desirable trait in wheat breeding. Several major QTLs controlling erect flag leaf from Zang1817 were detected, which could be used for optimizing plant architecture through molecular breeding after performing fine-mapping and cloning in the future. However, we have to consider their effects on the yield. For example, *QFLANG*-*4B* from Zang1817, which contributed to erect flag leaf, also leads to yield decrease.

The distribution for FLL, FLW, and FLA showed a continuous variation, indicating a quantitative genetic basis. The broad-sense heritability of FLL, FLW, FLA, and FLANG was 83, 88, 79, and 82%, respectively, indicating that flag leaf-related traits had a relatively high heritability. The FLANG was not a normal distribution in the RIL population. It might be due to the leaf angle significantly influenced by environment and cultivation conditions, and their variation in different environments is the largest among four flag leaf traits (Table 1). Furthermore, the FLANG may be controlled by several major QTLs (Cristaldo et al. 1992; De Carvalho and Qualset 1978). It has also been reported that flag leaf angle was under the control of approximately three genes (Joshi and Chand 2002).

Flag leaf posture and size is important in plant breeding, because it affects crop canopy morphology and photosynthetic efficiency. However, only a few studies reported on the genetic control of flag leaf traits in wheat. In this study, we detected seven QTLs for FLL. Two of these (*QFLL*-*2B* and *QFLL*-*3A*) were located in the intervals *Xbarc318*–*Xwmc344* and *Xcwem20*–*Xwmc532*, respectively; similar genetic regions were reported by Wu et al. (2015), and Fan et al. (2015). Several new FLL loci were identified on chromosomes 4B and 5A. Three QTLs for FLW were identified, two of which (*QFLW*-*4B.1* and *QFLW*-*4B.2*) were previously reported by Fan et al. (2015) to be at a similar genetic region on chromosome 4B. The QTL *QFLW*-*1B* might be a new, environmentally stable locus for FLW. Five QTLs for FLA were identified; three of these (*QFLA*-*2B*, *QFLA*-*4B.1*, and *QFLA*-*4B.2*) were previously reported by Wu et al. (2015) and Fan et al. (2015). Two new loci were detected on chromosome 5A, one of which (*QFLA*-*5A.1*) was environmentally stable. Eight QTLs for FLANG were detected, four of which (*QFLANG*-*1B.1*, *QFLANG*-*1B.2*, *QFLANG*-*6B.1*, and *QFLANG*-*6B.2*) were identified at similar regions by Wu et al. (2015). Four of these eight QTLs might be new loci, and of these, three (*QFLANG*-*3D*, *QFLANG*-*7B*, and *QFLANG*-*7D*) were environmentally stable.

In our study, two QTLs (*QFLL*-*4B.1* and *QFLL*-*4B.2*) for FLL were found to co-localize with QTL for FLA, FLW, and FLANG, consistent with the strong phenotypic correlations between these loci. We also found that the FLL QTL *QFLL*-*2B*, *QFLL*-*5A.2*, and *QFLL*-*5A.3* also co-localized with QTL for FLA (*QFLA*-*2B*, *QFLA*-*5A.1*, and *QFLA*-*5A.2*). Similarly, QTLs for FLANG (*QFLANG*-*1B.1* and *QFLANG*-*1B.2*) were detected with QTL for FLW on chromosome 1B. Given that QTL for flag leaf-related traits co-localized in similar regions, this indicates that these regions contain a major QTL with pleiotropic effects or multiple linked QTL. QTL mapping of yield-related traits using the same genetic population (ND3331/Zang1817) had been published by Liu et al. (2014). We also identified some regions that control not only flag leaf-related traits but also yield-related traits in this RIL population. For example, *QFLL*-*4B.1*, *QFLW*-*4B.1*, *QFLW*-*4B.2*, *QFLA*-*4B.1*, *QFLA*-*4B.2*, and *QFLANG*-*4B* were detected close (2–10 cM) to maker *Xbarc20*, where the QTLs of yield-related traits including PH, SL, SN, KN, GW, and TGW were also detected (Liu et al. 2014). The conditional QTL analysis revealed that FLL is responsible for FLA and FLANG, and FLA is also responsible for FLANG on chromosome 4B locus and flag leaf-related traits were also responsible for yield-related traits on chromosomes 2A, 4B, 6D, and 7A loci. Therefore, all these observations indicated the genetic interdependencies between flag leaf-related traits and yield-related traits.

In summary, we identified 15 stable QTL for flag leaf-related traits including FLL, FLW, FLA, and FLANG. The closely linked molecular markers in these loci have great potential value in marker-assisted selection to improve wheat flag leaf posture and size. Fine-mapping or cloning these major stable QTL and QTL regions with pleiotropic effects would advance our understanding of the underlying molecular mechanisms.

### **Author contribution statement**

JD and QS defined the research theme and supervised the project. KL and HX performed the experiments. GL contributed to genetic linkage map construction. PG and XZ contributed to phenotyping. HP contributed to QTL analysis. YY and ZN contributed to data analysis, interpretation, and presentation. All authors have read and approved the final version of the manuscript.

## Electronic supplementary material

Below is the link to the electronic supplementary material.

Supplementary material 1 (XLSX 42 kb)

Supplementary material 2 (XLSX 57 kb)
